# Disproportionate impact of the COVID-19 pandemic on immigrant communities in the United States

**DOI:** 10.1371/journal.pntd.0008484

**Published:** 2020-07-13

**Authors:** Eva Clark, Karla Fredricks, Laila Woc-Colburn, Maria Elena Bottazzi, Jill Weatherhead

**Affiliations:** 1 Department of Medicine, Section of Infectious Diseases, Baylor College of Medicine, Houston, Texas, United States of America; 2 Department of Medicine, Section of Health Services Research, Center for Innovations in Quality, Effectiveness, and Safety (IQuESt), Michael E. DeBakey VA Medical Center, Houston, Texas, United States of America; 3 Department of Pediatrics, National School of Tropical Medicine, Baylor College of Medicine, Houston, Texas, United States of America; 4 Section of Global and Immigrant Health, Department of Pediatrics, Texas Children’s Hospital, Baylor College of Medicine, Houston, Texas, United States of America; 5 Center for Vaccine Development, Department of Pediatrics, Texas Children’s Hospital, Baylor College of Medicine, Houston, Texas, United States of America; 6 Departments of Pediatrics and Molecular Virology & Microbiology, National School of Tropical Medicine, Baylor College of Medicine, Houston, Texas, United States of America; 7 Department of Biology, Baylor University, Waco, Texas, United States of America; Charles Sturt University, AUSTRALIA

In early 2020, a novel coronavirus (SARS-CoV-2) began to trickle through global communities, resulting in a pandemic of proportions not seen since 1918. In the US, while the disease caused by SARS-CoV-2, COVID-19, initially affected international travelers and their close contacts, it is now ravaging many disadvantaged communities. As in past pandemics, social and economic determinants will strongly influence susceptibility to and health outcomes of COVID-19; thus, it is predictable that low-income and vulnerable US populations will be disproportionately affected. Certain “hot spots” have already demonstrated high rates of COVID-19–related mortality in minority populations, particularly those of impoverished communities, likely due to increased prevalence of comorbid conditions as a result of unequal socioeconomic factors and inadequate access to timely healthcare [1–5]. We can anticipate similar outcomes in other vulnerable populations, particularly in immigrant communities, which have similar socioeconomic status and rates of comorbidities. With over 46.7 million immigrants currently living in the US, of which 11 million are undocumented [6], a socioeconomic perspective of the ongoing COVID-19 pandemic within the US immigrant community is necessary. Here, we will focus on the potential impact of COVID-19 on immigrant communities in the US, particularly those in Texas.

## Why is the COVID-19 pandemic likely to disproportionately affect US immigrants?

**Fig 1:**
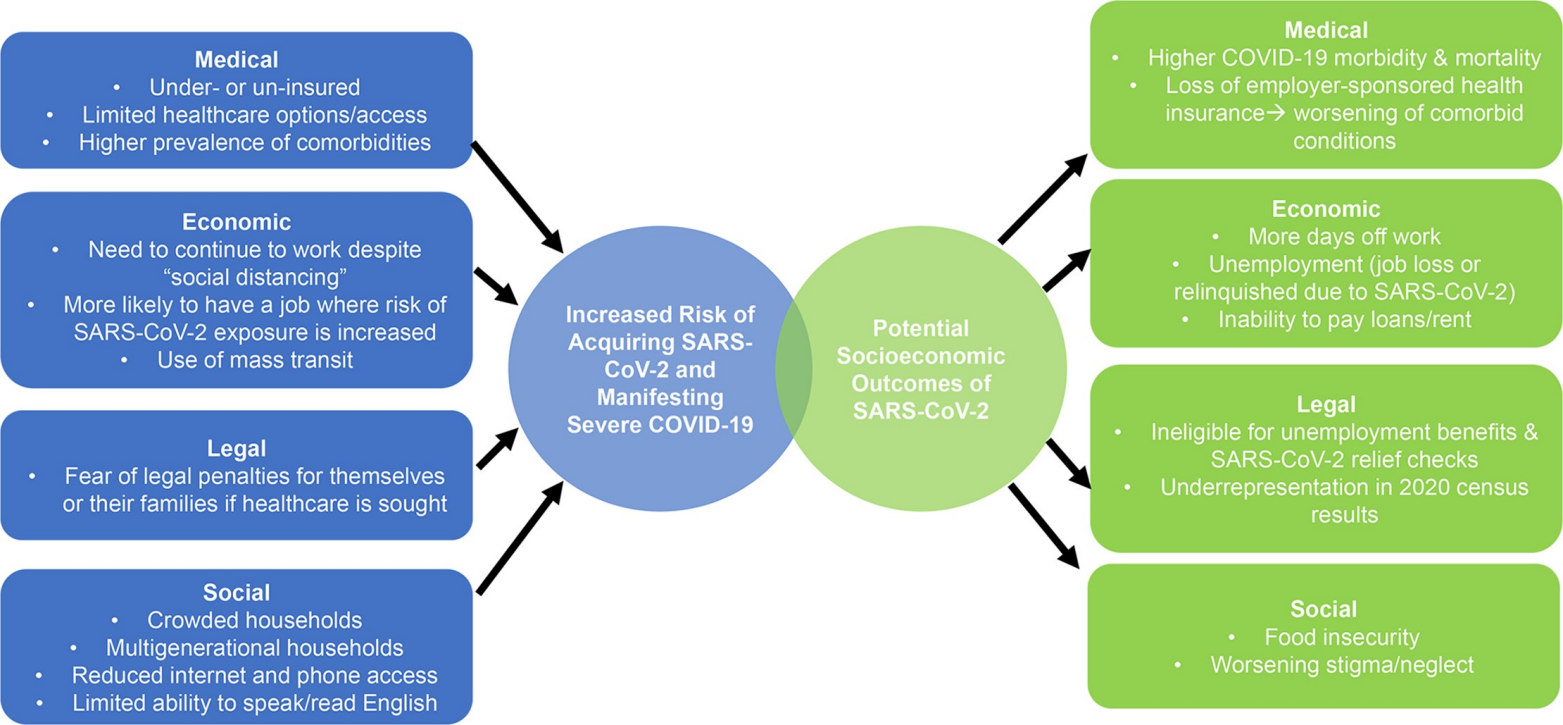
Risk factors and anticipated socioeconomic outcomes for the COVID-19 pandemic in vulnerable immigrant communities.

The intricacies of poverty, limited access to healthcare, and fear of legal repercussions place vulnerable immigrant communities within the US at high risk for acquiring SARS-CoV-2 and developing severe COVID-19 (Fig 1). Houston is an excellent example of a large, prosperous US city that is made up of (and depends upon) immigrants. Currently, there are an estimated 1.6 million immigrants (23.3% of the population) living in Houston, the majority of whom are from Mexico (40.2%), El Salvador (7.6%), Vietnam (5.9%), India (5.5%), and Honduras (3.6%). More than 500,000 of these immigrants (37.2%) are undocumented [7,8]. In Texas as a whole, an estimated 32% of undocumented immigrants live below the poverty level, and 64% are uninsured with limited options to meet their medical needs [8].

The lack of readily accessible, affordable healthcare [9,10] is particularly consequential during the COVID-19 pandemic. First, early diagnosis and monitoring of persons with COVID-19 is critical both to optimize the individual patient’s outcome and to prevent further community transmission. Many vulnerable immigrants are under- or uninsured [11] and thus depend upon Federally Qualified Health Centers (FQHCs), safety-net public health systems, or free clinics. These organizations are often underfunded, limiting their ability to provide testing, management, and follow-up services to their patients. Second, lack of access to preventive medicine leads to increased risk of underlying health conditions such as obesity, hypertension, and diabetes-—comorbidities that have been linked to more severe COVID-19 manifestations [9,12–15]. In a national evaluation of health conditions in immigrant populations, nearly a third (27.7%) of those from Mexico, the Caribbean, and Central America had hypertension, 71.5% had obesity, and 9.6% had diabetes [15], compared with the age-adjusted prevalence of 45.4%, 42.4%, and 8.2%, respectively, in the US general population [16]. However, within the US general population, these comorbidities tend to be higher in minority groups compared to whites; for instance, while the prevalence of diabetes in the US general population was 8.2% overall, it was 12.5% for people of Hispanic origin, 11.7% for non-Hispanic Blacks, and 7.5% for non-Hispanic Whites [17]. Third, depending on their mode of entry into the US, many immigrants may be at risk for excessive stress related to poverty, trauma, and poor social support, which leads to mental health conditions such as post-traumatic stress disorder, depression, and anxiety [18]. These psychological stressors may be worsened during a pandemic, certainly for those with limited healthcare resources, high risk of job loss, or high risk of SARS-CoV-2 exposure.

Regarding risk of SARS-CoV-2 exposure, many immigrants are at increased risk both because their economic situation requires continuation of work despite “social distancing” and “stay-at-home” recommendations and because the types of jobs most commonly worked by immigrants often require face-to-face interactions. Immigrants make up more than 20% of the Texas work force and are employed most commonly in construction, accommodation, food services, healthcare, and manufacturing industries [8,19]; these are “essential” professions that do not lend themselves to working from home [20]. In addition, immigrants who continue working are more likely to use public mass transit to get to their jobs, which further increases their risk of SARS-CoV-2 exposure [21].

In the home, immigrants are more likely to live in large, multigenerational family groups or with multiple roommates. Nearly 29% of Asian, 27% of Hispanic, and 26% of Black Americans live in multigenerational households, a practice that is particularly common in those who are foreign-born [22]. Logically, if one person living in a crowded home is infected with SARS-CoV-2, their cohabitants, including elderly and immunosuppressed ones, will likely be exposed as well. Finally, recent immigrants and their families are less likely to have cell phones or internet access [23] and to speak and read English; in Texas, for example, approximately 50% of undocumented immigrants lack English proficiency [8]. Consequently, immigrant communities with limited English skills may be less likely to receive and understand public health messages, warnings, and updates.

## What is the potential socioeconomic impact of the COVID-19 pandemic on US immigrants?

One of the ways US immigrants play a significant role in the US economy is by paying federal, state, and local taxes. In 2018, immigrants in Texas paid 38.6 billion dollars in taxes, of which undocumented immigrants assigned Individual Taxpayer Identification Numbers (ITINs) contributed an estimated $4.2 billion [24]. Despite this, ITIN holders do not qualify for COVID-19 federal economic relief through the Coronavirus Aid, Relief, and Economic Security (CARES) Act. Families with mixed immigration status who file jointly, such as undocumented adults with children or spouses who are US citizens, are also excluded because all individuals included in a tax return must have valid Social Security numbers to be eligible [25]. As such, despite paying into the US economy and experiencing equal, if not more severe, consequences from the COVID-19 pandemic, many immigrants will not receive any COVID-19–related economic relief from the US government.

Thus, there is much concern that the COVID-19 pandemic will result in particularly high rates of unemployment and financial strain within immigrant communities [26]. Between February 2020 and April 2020, the unemployment rate for immigrant women increased from 4.3% to 18% and for immigrant men from 3% to 15.3%, while for US-born women, it changed from 3.3% to 15.3%, and for US-born men, it increased from 4.3% to 12.8% [27]. Because undocumented immigrants are ineligible for national unemployment benefits in addition to CARES Act benefits, job loss or reduced work hours due to the COVID-19 pandemic may lead to significantly decreased financial reserve in immigrant households. For the 57% of immigrants who have private insurance [1], loss of a job could also mean loss of health insurance for the employee and their family, leading to further difficulty accessing healthcare. Those who are able to retain their jobs, as discussed above, may work in sectors not amenable to working from home or that do not permit sick leave [20,28]. Higher numbers of uninsured immigrants combined with those working in high SARS-CoV-2 exposure risk jobs will undoubtably result in increased COVID-19–related morbidity and mortality in immigrant communities. Additionally, in the context of extended school closing as a result of COVID-19, many parents have limited childcare options, putting additional financial, health, and social pressure on families.

As the COVID-19 pandemic causes instability in global supply chains, concern for worsening food insecurity is growing in many disadvantaged communities. Immigrants are at particularly high risk, especially those who have resided in the US for less than 5 years [29]. This may be because immigrant families newly arrived in the US have more significant language barriers and less exposure to a stable education system and jobs than those who have lived in the US for longer periods of time. However, even immigrant families who have lived in the US for more than 10 years are at higher risk of food insecurity than US-born households. According to one study, more than 30% of children born to Mexican and Central American immigrants are already subject to food insecurity [29]. Additionally, the federal government stipulates that adults (with some exceptions) with legal permanent resident status (i.e., green card holders) must wait 5 years before they can apply for the Supplemental Nutrition Assistance Program (SNAP) [30]. Furthermore, despite being eligible for SNAP, low-income US citizen children with immigrant parents have decreased utilization of this benefit in recent years [31]. Although children with green cards are not subject to the 5-year waiting period and may qualify for SNAP along with low-income US citizen children, studies have shown that eligible children of ineligible parents are less likely to participate in assistance programs [32]. This has become increasingly apparent in recent years because of concern over the “Public Charge” rule (implemented on February 24, 2020), which limits the ability of immigrants to adjust to legal permanent resident status if they have used certain public benefits. The fear of deportation and chilling effect of this rule have led many immigrant families to forgo participation in all federal assistance programs, including nutrition assistance, even if they are eligible and not subject to a public charge determination. The factors driving this downward trend will likely also prevent many eligible immigrant families from applying for Pandemic Electronic Benefit Transfer (EBT), a provision of the Families First Coronavirus Response Act allowing states to provide money to families whose children were receiving free or reduced cost meals through their schools. This would have a far-reaching impact because, unlike SNAP, Pandemic EBT is available to children regardless of immigration status. Expansion of “food deserts” as a result of limited transportation options and restaurant restrictions, reduced grocery store supply, and diminished resources in food banks may further limit food availability in at-risk immigrant communities [33–35].

Finally, due to the implementation of recent immigration policies such as the “Public Charge” rule, the utilization of available health resources among immigrants and their families has effectively decreased as a result of widespread fear of immigration enforcement and/or concern that using these services would impair their success of future naturalization. In addition, mounting health, psychosocial, and financial concerns—together with fears of legal exposure—may inhibit immigrant participation in the ongoing 2020 census data collection [25]. In the long term, inadequate enumeration of the US immigrant populations will manifest as decreased funding for sorely needed health, education, and socioeconomic programs in many disadvantaged communities.

## Déjà vu: Comparing COVID-19 to H1N1

**Fig 2:**
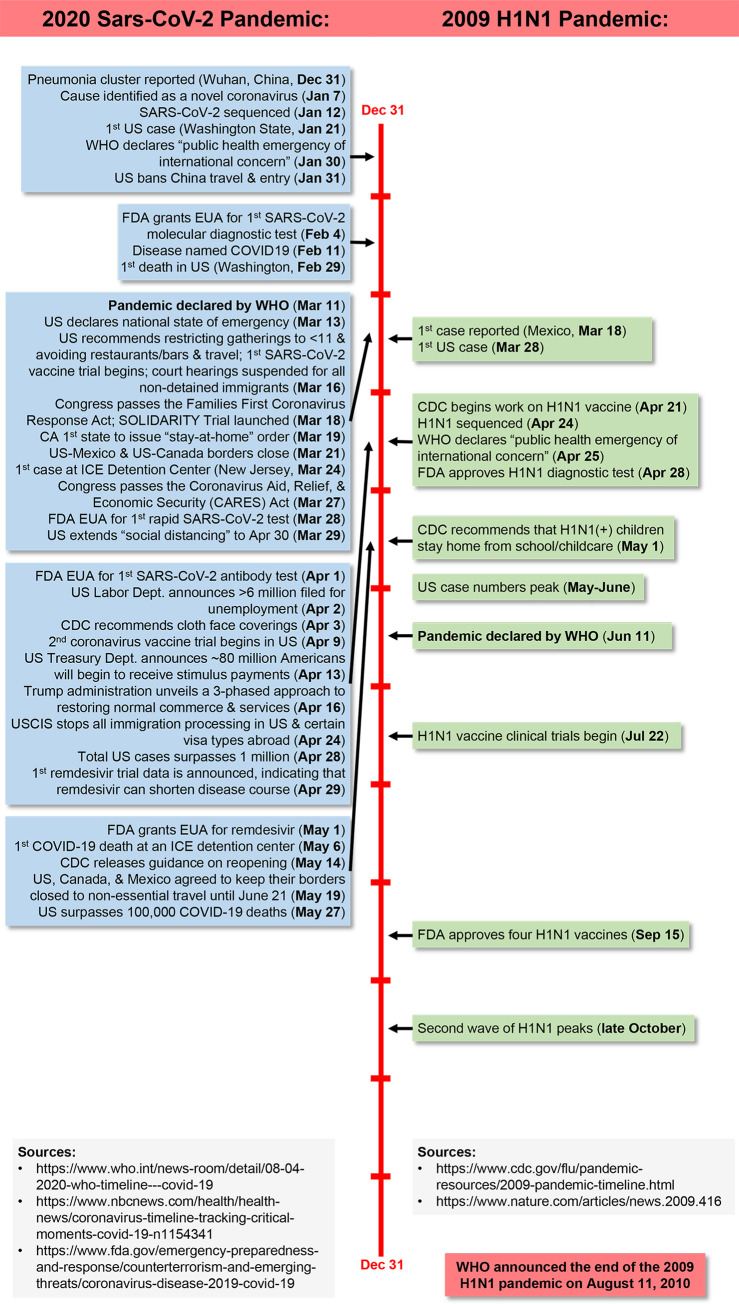
Comparison of COVID-19 and 2009 H1N1 influenza pandemic timelines in the context of events affecting vulnerable immigrant communities in the US. CDC, US Centers for Disease Control and Prevention; EUA, emergency use authorization; FDA, US Food and Drug Administration; ICE, US Immigration and Customs Enforcement; USCIS, US Citizenship and Immigration Services; WHO, World Health Organization.

The COVID-19 pandemic is certainly not the first pandemic to reveal underlying health disparities. Most recently, the 2009 H1N1 influenza pandemic provided opportunity to understand health inequalities in vulnerable US populations that parallel those emerging in the current COVID-19 pandemic (Fig 2). Both suggest poor health and economic outcomes in disadvantaged populations such as at-risk immigrants.

In the spring of 2009, Mexico reported a number of cases of influenza-like illness caused by a novel H1N1 virus. This virus disseminated rapidly, and the World Health Organization (WHO) declared H1N1 influenza a pandemic in June 2009 [36]. The final burden of H1N1 disease in the US was estimated to be approximately 60.8 million cases, with more than 274,000 hospitalizations and more than 12,000 deaths [37]. Like SARS-CoV-2, H1N1 frequently caused severe lung injury [38]. Specific risk factors for severe H1N1 disease included obesity, pregnancy, immunosuppression, lung disease, HIV infection, poverty, and lack of access to healthcare [39–41]. Additionally, factors such as limited access to and use of preventive medical care [42], large household sizes [28], difficulties complying with work-from-home directives (even when ill) because of the need to work [20,28], and reliance on public transportation [21] placed immigrants at high risk of H1N1. Surveillance case reports during the 2009 H1N1 pandemic were disproportionately high among all disadvantaged groups, and the Hispanic population specifically was noted to have increased influenza-associated hospitalization and pediatric mortality [43]. The disproportionate effect of the H1N1 pandemic on Spanish-speaking Hispanics may have occurred because of increased risk of H1N1 exposure and greater disparity in access to healthcare compared with other disadvantaged groups [28]. However, there are sparse data on outcomes for other immigrant populations during the H1N1 pandemic because of limited surveillance. In addition to clinical outcome inequalities, the H1N1 pandemic exemplified the disparities in pandemic preparedness, response, and recovery for disadvantaged populations, including immigrant communities [21]. Unfortunately, since the H1N1 pandemic, health disaster preparedness for immigrant communities has largely remained inadequate.

## Conclusions and next steps

SARS-CoV-2 has severely impacted our global community, placing marginalized populations at high risk of contracting the virus and of developing severe COVID-19. As we learned from the H1N1 pandemic, it is imperative that we act urgently to support disadvantaged communities during this COVID-19 health and economic crisis. Taking action at local, state, and national levels to improve healthcare access as well as economic and legal protections for immigrant communities is critical [44]. Acutely, healthcare facilities should be designated as locations where immigration enforcement is prohibited. Such action will decrease the fear of seeking healthcare services. For those states that have not already done so, opting into Medicaid expansion would increase health insurance coverage for more low-income adults, including documented immigrants. Additionally, states should change their eligibility criteria for the Children’s Health Insurance Program (CHIP) to allow all children—regardless of immigration status—to be considered, thus increasing the number of immigrant children with healthcare coverage. Future COVID-19–related relief packages should include vulnerable immigrant groups and improve the availability of health services through the expansion of safety-net health systems in all disadvantaged communities [45]. Testing for SARS-CoV-2 should be made widely available, easily accessible, and free. Policy changes to prevent or mitigate devastating healthcare costs for uninsured patients with COVID-19 must be instituted. In the long term, improving primary care resources to diagnose, treat, and control comorbidities in high-risk populations may reduce poor outcomes in vulnerable immigrant communities during future pandemics. Additionally, measures to create and maintain safe employment opportunities would help to relieve immigrants’ economic burden while not increasing their exposure risk. Further, developing tools to rapidly disperse culturally and linguistically appropriate public health messages to at-risk immigrant communities will improve health education, preparedness, and response time. The care of disadvantaged communities—including immigrant populations—in the US must be prioritized to reduce the devastating, inequitable health and financial costs repeatedly and predictably accrued by immigrant populations during epidemic disease outbreaks.

**Policy recommendations to lessen the impact of the SARS-CoV-2 pandemic on US immigrants**

- Expand Medicaid in every state to cover more low-income adults.
- Eliminate immigration status requirements for children when assessing their eligibility for the Children’s Health Insurance Program (CHIP).
- Pass legislation stating that Immigration and Customs Enforcement (ICE) cannot conduct any operations at or near healthcare facilities.
- Fund SARS-CoV-2 testing and COVID-19 treatment for all uninsured individuals, regardless of immigration status.
- Include immigrants who have an Individual Taxpayer Identification Number (ITIN) and their families in economic relief packages (not just those with a Social Security number).
